# How tourism experiences relate to eWOM intention: the roles of self-expansion and place attachment in ancient town tourism

**DOI:** 10.3389/fpsyg.2026.1892209

**Published:** 2026-08-26

**Authors:** Weifang Zhan, Chengpeng Lin

**Affiliations:** 1 Department of Hotel & Convention Management, Honam University, Gwangju, Republic of Korea; 2 Department of Business Administration, Kangnam University, Yongin, Republic of Korea

**Keywords:** experience economy, self-expansion, place attachment, electronic word-of-mouth intention, ancient towns

## Abstract

Why are certain tourism experiences more strongly associated with tourists’ self-expansion, place attachment, and destination advocacy than others? Drawing on self-expansion theory, this study examines the psychological processes through which experiential consumption in ancient town tourism is associated with electronic word-of-mouth (eWOM) intention. Integrating the experience economy framework, self-expansion theory, place attachment theory, and the stimulus–organism–response framework, we propose that tourism experiences are associated with eWOM intention indirectly through self-expansion and place attachment. Survey data were collected from 410 tourists who had recently visited representative ancient towns in the Jiangnan region of China and analyzed using structural equation modeling. The results indicate that educational, entertainment, and escapist experiences are positively associated with self-expansion, whereas aesthetic experience is not significantly associated with self-expansion. In addition, self-expansion is positively associated with both place dependence and place identity, and both dimensions of place attachment are positively associated with eWOM intention. Bootstrapping analyses further support significant indirect associations between several tourism experience dimensions and eWOM intention through self-expansion and place attachment. These findings extend previous tourism research by providing additional evidence that self-expansion is associated with tourists’ place-related attachment and online advocacy. More broadly, the findings contribute to personality and social psychology by suggesting that tourism experiences are associated with self-concept enrichment and place-related identity, which may be reflected in online advocacy as a form of identity expression.

## 1 Introduction

Tourism experiences are increasingly recognized for their potential to relate to psychological enrichment, self-development, and identity-related processes. Beyond simple leisure, contemporary tourism provides a context for individuals to encounter new perspectives and engage in self-relevant meaning-making that transcends immediate consumption (Li et al., 2024). This shift reflects an experiential turn where tourists seek encounters that are personally significant and capable of enriching their self-concepts. From a personality and social psychology perspective, tourism may be associated with changes in self-perception as individuals integrate novel perspectives and social interactions into their evolving self-structures. Consequently, understanding the psychological mechanisms through which tourism experiences become personally significant has become a salient area of inquiry.

Among various cultural contexts, ancient town tourism offers a suitable environment for examining these psychological processes. Characterized by historical authenticity and cultural symbolism, ancient towns allow tourists to engage with destinations beyond functional service interactions (Yu et al., 2020). Such culturally embedded environments may encourage reflection and identity-related experiences through encounters with traditional architecture and local customs. These characteristics align with the framework of transformative tourism, which emphasizes experiences associated with personal growth and changes in how individuals perceive their place-related identity. In China, the ancient water towns of the Jiangnan region are iconic cultural destinations; however, the increasing homogenization of infrastructure and services has become a concern (Chen et al., 2023). As destinations become more similar, providing enjoyable services alone may be insufficient to relate to tourists’ voluntary engagement in online advocacy.

While previous research has established that memorable tourism experiences are associated with satisfaction and loyalty (Oh et al., 2007; Sihombing et al., 2024), and may be linked to destination attachment (Cardinale et al., 2016), the predominantly service-oriented perspective often overlooks underlying psychological mechanisms. Specifically, less attention has been devoted to how tourism experiences relate to self-concept enrichment, which may, in turn, be associated with voluntary online communication. Addressing this gap is essential as tourism increasingly functions as a medium for identity expression and psychological growth.

The significance of these processes is heightened in the digital era. Social media has shifted tourists from passive recipients to active participants who share experiences via electronic word-of-mouth (eWOM) (Hennig-Thurau et al., 2004). Rather than being a mere consequence of satisfaction, online sharing may represent a form of identity expression, allowing individuals to communicate personally meaningful experiences to a broader audience. Despite its importance, research on ancient town tourism has focused heavily on revisit intentions, with limited exploration of the psychological processes linked to the willingness to share experiences online (Zhou et al., 2024).

To address these limitations, the present study adopts Self-Expansion Theory as a primary lens. This theory suggests that individuals are motivated to expand their sense of self by acquiring new perspectives, identities, and resources through interactions with their environment (Aron et al., 2013). In tourism, culturally immersive encounters may be associated with perceived self-expansion and self-concept enrichment. These psychological states may further relate to place-related identity and subsequent eWOM intentions. Building upon the Experience Economy framework, Place Attachment Theory, and the Stimulus–Organism–Response (S–O–R) model, this study examines an integrated model to clarify the associations among tourism experiences, self-expansion, place attachment, and eWOM.

This study positions itself as an extension of existing work rather than a definitive departure. It seeks to clarify the associations between meaningful tourism experiences, identity-related outcomes, and post-consumption advocacy. Specifically, the findings offer the following theoretical insights. First, this work extends the Experience Economy framework by exploring its association with perceived self-expansion. Second, it adds to the literature on place attachment by examining how self-expansion relates to both place dependence and place-related identity. Third, it contributes to the eWOM literature by suggesting that online advocacy may reflect identity expression stemming from psychologically meaningful experiences. Collectively, these findings provide an empirical basis for understanding the associations between tourism encounters and psychological development.

## 2 Literature review

### 2.1 Tourism experience in ancient town

Ancient towns are culturally embedded environments where historical architecture, traditional lifestyles, and symbolic meanings intersect within social spaces (Su et al., 2021). Unlike highly commercialized destinations that emphasize hedonic consumption, ancient towns offer contexts for tourists to engage with culturally authentic environments and historically significant places (McCartney and Chen, 2020). In such settings, tourists often transition from passive observers to active participants in culturally and emotionally immersive experiences.

The tourism experience is conceptualized as a multidimensional psychological response arising from interactions between tourists and destinations (Oh et al., 2007). As consumption shifts toward an experiential focus, tourists increasingly seek encounters that are personally significant and capable of enriching their self-concepts rather than merely providing functional services (Sundbo and Sørensen, 2013). Recent research suggests that these meaningful experiences may be associated with transformative learning, self-reflection, and identity-related processes, highlighting the role of tourism in psychological development. Within this context, the Experience Economy framework (Pine and Gilmore, 1998) provides a foundational approach for examining experiential consumption.

According to this framework, tourism experiences are classified into four dimensions based on the level of participation and immersion: education, entertainment, aesthetics, and escapism. Educational experiences involve cognitive engagement through learning and knowledge acquisition, while entertainment emphasizes sensory stimulation and pleasure. Aesthetic experiences emerge through the appreciation of symbolic atmospheres and physical environments. Escapist experiences involve immersion that allows individuals to temporarily explore alternative perspectives and “possible selves” beyond their ordinary identities (Oh et al., 2007). Empirical studies have indicated that these dimensions relate to tourists’ emotional states, attachment, and behavioral intentions across diverse contexts, including heritage and rural tourism (Loureiro, 2014; Jiang et al., 2024).

Despite the accumulation of research on tourism experiences, many studies have explained post-consumption behaviors primarily through evaluative constructs such as service quality, satisfaction, and perceived value. Relatively less focus has been placed on the underlying psychological mechanisms through which these experiences relate to self-expansion and changes in self-structures. This gap is salient in ancient town tourism, where culturally symbolic environments may provide experiences that transcend temporary enjoyment. Self-expansion theory offers a valuable psychological perspective for explaining how tourism encounters are associated with perceived growth and identity-related development. Accordingly, the present study extends the Experience Economy perspective by examining how specific experiential dimensions are associated with tourists’ self-expansion, thereby providing a psychological framework for understanding the links between tourism experiences, place attachment, and online advocacy.

### 2.2 Self-expansion theory

Self-expansion theory suggests that individuals are motivated to enrich their self-concepts by acquiring new perspectives, identities, and resources through significant experiences and social interactions (Aron et al., 2013). Rather than viewing the self as a static entity, this theory conceptualizes identity as a dynamic structure that evolves as individuals integrate novel experiences into their self-perceptions. From a personality and social psychology perspective, self-expansion represents a psychological process through which the integration of novel encounters is associated with perceived personal growth and self-concept enrichment. Initially developed to explain interpersonal relationships, the theory has been extended to consumer behavior and experiential consumption, where meaningful encounters relate to individuals’ perceptions of identity enhancement (Reimann and Aron, 2014).

In consumption contexts, self-expansion is thought to occur when experiences offer opportunities to acquire new knowledge, perspectives, and symbolic meanings that are subsequently incorporated into the self-structure. Empirical work has indicated that immersive consumption—such as luxury or virtual reality experiences—relates to self-expansion by providing perceptions of novelty and identity enrichment (Barreda-Ángeles and Hartmann, 2022). Tourism, in particular, offers a context for self-expansion, as it involves voluntary encounters with unfamiliar cultures and environments that may encourage reflection and learning (Shen et al., 2024). These experiences are associated with how individuals perceive themselves and interpret personally significant travel encounters beyond immediate enjoyment.

Ancient town tourism offers a suitable context for examining self-expansion due to the immersion in culturally symbolic environments that differ from everyday routines. Such settings provide opportunities for learning and identity-related reflection through encounters with historical narratives and traditional lifestyles. From the Experience Economy framework, each dimension may relate to self-expansion through distinct psychological pathways. Educational experiences are associated with the acquisition of cultural knowledge, which may relate to perceived personal competence. Entertainment experiences relate to positive affect, which is often associated with psychological vitality and an openness to new experiences. Aesthetic experiences involve immersion in symbolic environments, which may be linked to the internalization of meanings associated with heritage sites. Escapist experiences allow individuals to temporarily distance themselves from routine roles and explore “possible selves” (Oh et al., 2007). Collectively, these dimensions are expected to be associated with perceived self-expansion by enriching both psychological resources and identity-related experiences.

While recent research has applied Self-Expansion Theory to heritage tourism (Chen et al., 2025), the literature has focused more on destinations as symbolic brands than on the specific experiential dimensions associated with self-expansion. Furthermore, there is a need for a clearer understanding of the psychological mechanisms linking tourism experiences with identity-related outcomes. Building upon existing work, the present study examines self-expansion as a psychological mechanism linking the dimensions of the experience economy with subsequent psychological responses. Accordingly, the following hypotheses are proposed:

*H1*: The four dimensions of the experience economy are positively associated with tourists’ self-expansion in ancient town tourism.

*H1a*: Educational experiences are positively influence tourists’ self-expansion.

*H1b*: Entertainment experiences are positively influence tourists’ self-expansion.

*H1c*: Escapist experiences are positively influence tourists’ self-expansion.

*H1d*: Aesthetic experiences are positively influence tourists’ self-expansion.

### 2.3 Place attachment

Place attachment refers to the emotional and psychological bond that individuals develop with specific environments through their experiences and interactions (Hernández et al., 2007). In tourism research, place attachment is recognized as a key construct for understanding how tourists establish emotional connections with destinations, which may be associated with subsequent behavioral intentions (Lee, 2012). Rather than a merely functional evaluation, place attachment reflects the extent to which a destination acquires personal and identity-relevant significance through lived experiences. As tourists engage with meaningful places, these destinations may become integrated into their self-concepts and autobiographical memories, relating to a sense of enduring psychological connection.

The conceptualization of place attachment typically consists of two dimensions: place dependence and place identity (Williams and Vaske, 2003). Place dependence reflects a functional attachment based on the perceived ability of a destination to satisfy experiential needs more effectively than alternative settings (Hammitt et al., 2006). In contrast, place-related identity reflects a symbolic and emotional attachment where individuals psychologically associate a destination with their self-structures and memories (Kyle et al., 2004). In experiential tourism, destinations may become incorporated into tourists’ self-perceptions when encounters provide deep emotional immersion and symbolic meaning.

The Stimulus–Organism–Response framework provides a theoretical foundation for examining these psychological processes. According to the S–O–R model, environmental stimuli are associated with individuals’ internal psychological states, which, in turn, relate to behavioral responses (Şahin and Kılıçlar, 2023). In this study, the four dimensions of the experience economy are examined as environmental stimuli, while self-expansion and place attachment represent the internal psychological mechanisms associated with subsequent electronic word-of-mouth (eWOM) intentions.

Previous studies have indicated that experiential consumption is associated with place attachment. For instance, Cardinale et al. (2016) noted that experiential dimensions relate to tourists’ attachment to wine destinations, while Kastenholz et al. (2020) suggested that immersive encounters are linked to emotional bonding in rural tourism. In culturally symbolic settings like ancient towns, these experiential dimensions may relate to place dependence by enhancing the perceived uniqueness of the destination. Concurrently, meaningful experiences may be associated with place identity by allowing tourists to integrate destination-related meanings into their evolving self-concepts.

From a Self-Expansion Theory perspective, individuals who perceive identity enrichment are more likely to develop psychological bonds with the environments associated with those experiences. As tourism encounters become incorporated into the self-concept, destinations may acquire symbolic significance, relating to both place dependence and place-related identity. Empirical work has suggested associations between self-expansion and attachment formation in various consumption contexts (Chen et al., 2025). Accordingly, self-expansion is examined here as a psychological mechanism linking tourism experiences with place attachment. The following hypotheses are proposed:

*H2*: The experience economy of the ancient towns positively associated with tourists’ place attachment.

*H2a-d*: Educational, entertainment, escapist, and aesthetic experiences are positively associated with place dependence.

*H2e-h*: Educational, entertainment, escapist, and aesthetic experiences are positively associated with place identity.

*H3*: Self-expansion is positively associated with tourists’ place attachment.

*H3a*: Self-expansion is positively associated with place dependence.

*H3b*: Self-expansion is positively associated with place identity.

### 2.4 Electronic word of mouth intention

Electronic word-of-mouth encompasses statements regarding products, services, or destinations shared through digital communication platforms (Hennig-Thurau et al., 2004). Unlike traditional face-to-face communication, eWOM enables tourists to share travel encounters with broad audiences via social media and online review platforms (Xiang and Gretzel, 2010). As tourism consumption has become increasingly digitized, eWOM has emerged as a salient factor in destination image and tourists’ decision-making (Pandey and Sahu, 2020). Consequently, understanding the factors associated with positive eWOM is important for destination competitiveness and sustainability.

From a personality and social psychology perspective, eWOM represents more than a post-consumption evaluation. Beyond information sharing, online communication serves as a medium for identity expression and online advocacy, reflecting experiences that have become integrated into the self-concept. Tourists who perceive destinations as psychologically significant are more likely to engage in voluntary advocacy by sharing identity-relevant travel experiences with others (Sparks and Browning, 2011). In this view, eWOM reflects a willingness to communicate encounters that have acquired personal and symbolic significance.

Empirical evidence suggests associations between place attachment and tourists’ word-of-mouth behaviors. For instance, Turki and Amara (2017) noted that emotional attachment relates to tourists’ willingness to recommend destinations, while Sop and Kervankıran (2023) indicated that both place dependence and place-related identity are associated with word-of-mouth intentions. Consistent with the Stimulus–Organism–Response framework, place attachment is examined as an internal psychological state associated with subsequent behavioral responses, such as eWOM (Pandey and Sahu, 2020). Accordingly, tourists who develop emotional and symbolic bonds with destinations are expected to exhibit a greater tendency to engage in online advocacy. Based on these associations, the following hypotheses are proposed:

*H4*: Place attachment is positively associated with tourists’ eWOM intention in ancient town tourism.

*H4a*: Place dependence is positively associated with tourists’ eWOM intention.

*H4b*: Place identity is positively associated with tourists’ eWOM intention.

**Figure 1:**
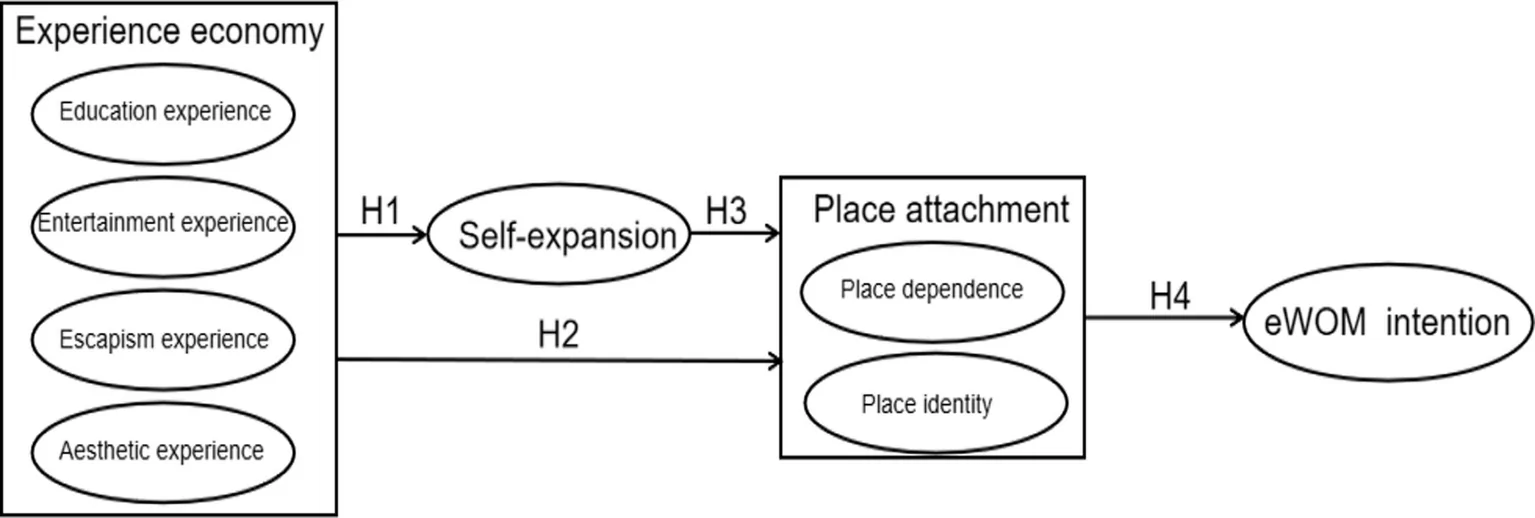
Conceptual model.

The conceptual framework of the present study is presented in Figure 1.

## 3 Methodology

### 3.1 Survey instrument

The Jiangnan region, characterized by its well-preserved ancient water towns, is a significant context for heritage tourism in China (Chen et al., 2023). According to the 2024 China Ancient Town Tourism Development Report, destinations such as Wuzhen, Xitang, Nanxun, Zhouzhuang, and Tongli are widely recognized as representative cultural tourism sites. These locations were selected as the research context to provide a suitable environment for examining the proposed psychological associations.

Data were collected through an online questionnaire administered via Wenjuanxing, a professional survey platform in China, between June 1 and June 15, 2025. A self-administered approach was employed using survey links and QR codes. The questionnaire comprised three sections: (1) an introduction detailing the study’s purpose and ethical considerations, (2) measurement items adapted from validated scales, and (3) respondents’ demographic information.

Ethical protocols were strictly followed; participants were informed of the voluntary nature of the study and their right to withdraw at any time. Electronic informed consent was obtained from all respondents prior to data collection. To ensure data relevance, a screening question confirmed that respondents had visited at least one ancient water town in the Jiangnan region within the previous 6 months.

Measurement items for the Experience Economy, Self-Expansion, Place Attachment, and eWOM intention were adapted from established scales to fit the ancient town context. The Experience Economy scale was adapted from Oh et al. (2007) and Lee et al. (2020), focusing on the four experiential dimensions. Self-expansion and place attachment items were based on Chen et al. (2025), reflecting perceived growth and emotional bonding. The eWOM intention scale, reflecting voluntary online advocacy, was adapted from Pandey and Sahu (2020) and Rasoolimanesh et al. (2021). All constructs were measured using a five-point Likert scale ranging from 1 (strongly disagree) to 5 (strongly agree).

### 3.2 Respondents’ demographic profile

A total of 450 responses were collected. Following data screening, 40 questionnaires were excluded due to incomplete responses, careless answering patterns, or insufficient completion time (less than 4 min). Consequently, 410 valid responses were retained for analysis.

Data were analyzed using IBM SPSS 27 and AMOS 26. The analytical procedure included descriptive statistics, reliability and validity assessments (CFA), and structural equation modeling (SEM) to examine the associations among the constructs.

Descriptive analysis of the sample (*N* = 410) indicated a slightly higher proportion of female respondents (54.9%). The 18–35 age group represented the largest segment, comprising 73% of the sample. Over half of the respondents were married (55.6%), and 43.9% held a bachelor’s degree. Students (33.7%) and employees (29.8%) were the most prominent occupational groups. Regarding income, nearly 40% of the participants reported a monthly income below 3,000 yuan (see Table 1 for detailed demographics).

**Table 1:** Socio-demographic characteristics of respondents.

Profile	Frequency	Percent (%)
Sex		
Male	185	45.1
Female	225	54.9
Age		
18–25	138	33.7
26–35	161	39.3
36–45	64	15.6
46–55	37	9
56 and above	10	2.4
Marriage status		
Single	182	44.4
Married	228	55.6
Education level		
Junior high school graduate or less	38	9.3
High school graduation	55	13.4
Junior college enrollment/graduation	78	19
University enrollment/graduation	180	43.9
Postgraduate or above	59	14.4
Occupation		
Employee	122	29.8
Professional	45	11
Freelancer	44	10.7
Student	138	33.7
Unemployed	27	6.6
Other	34	8.3
Monthly income		
Less than RMB3000	158	38.5
RMB3001–5000	67	16.3
RMB5001–8000	75	18.3
RMB8001–14000	60	14.6
More than RMB 14000	50	12.2

## 4 Results

### 4.1 Common method bias test

Common method bias (CMB) may be a concern in studies where data are collected from a single source using self-reported measures. To assess the potential presence of CMB, Harman’s single-factor test was performed using IBM SPSS 27. The exploratory factor analysis identified eight factors with eigenvalues greater than 1, with the first unrotated factor accounting for 47.939% of the total variance. As this value remains below the 50% threshold (Podsakoff et al., 2003), CMB does not appear to be a major concern in the present data.

To further mitigate potential bias, several procedural remedies were implemented. Participation was voluntary and anonymous, and respondents were informed that there were no correct or incorrect answers. Additionally, measurement items were adapted from validated scales and organized into separate sections to minimize respondents’ tendency to infer relationships between constructs (Podsakoff et al., 2003). Nonetheless, given the cross-sectional and self-reported nature of the data, the possibility of common method variance is acknowledged and considered in the interpretation of the findings.

### 4.2 Reliability and validity testing

The reliability of the measurement scales was assessed using Cronbach’s alpha (Table 2). All alpha values exceeded the recommended 0.70 threshold (Nunnally and Bernstein, 1994), suggesting acceptable internal consistency.

**Table 2:** Results of confirmatory factor analyses.

Constructs	Statement	Label	loading	Cronbach α
Education experience	I learned a lot from this visit to the ancient town.	EDUE1	0.847	0.747
	This ancient town travel experience was highly educational for me.	EDUE2	0.802	
	I acquired a great deal of knowledge during this ancient town visit.	EDUE3	0.761	
Entertainment experience	The process of visiting the ancient town was very enjoyable.	ENTE1	0.742	0.634
	There were many fun and interesting things to see during my ancient town visit.	ENTE2	0.700	
	The ancient town tourism experience offered entertaining content.	ENTE3	0.559	
Escapism experience	Visiting the ancient town made me feel like I was living in a different time or place.	ESCE1	0.787	0.705
	The ancient town trip helped me completely forget about everyday worries.	ESCE2	0.779	
	During the ancient town visit, I felt fully detached from my real-life routine.	ESCE3	0.778	
Aesthetic experience	The design of the ancient town is very appealing to me.	AESE1	0.845	0.642
	The overall environment of the ancient town shows great attention to detail.	AESE2	0.758	
	The atmosphere of the ancient town provided me with sensory pleasure.	AESE3	0.697	
Self-expansion	This ancient town tourism experience broadened my horizons.	SE1	0.748	0.627
	This ancient town tourism experience gave me a deeper understanding of many things.	SE2	0.676	
	This trip helped me build up my knowledge about the ancient town and its culture.	SE3	0.642	
Place dependence	Ancient towns are my favorite places to engage in travel activities.	PD1	0.737	0.627
	Compared to other destinations, visiting ancient towns makes me feel more satisfied.	PD2	0.735	
	For me, ancient towns are irreplaceable travel destinations.	PD3	0.659	
Place identity	I feel that ancient towns have become a part of my life.	PI1	0.713	0.649
	I feel deeply emotionally attached to ancient towns.	PI2	0.692	
	Ancient towns hold significant meaning for me.	PI3	0.674	
Electronic word of mouth intention	I would use my personal social media to speak positively about ancient towns to friends and family.	eWOM1	0.782	0.614
	I would post on social media or my personal website about positive tourism in the ancient towns.	eWOM2	0.738	
	I would like to update social media or my personal website with positive content related to ancient town tourism in the future.	eWOM3	0.694	

Construct validity was initially examined through exploratory factor analysis (EFA). The results indicated that the eight factors accounted for 79.585% of the cumulative variance. Bartlett’s test of sphericity was significant (*p* < 0.001), and the Kaiser–Meyer–Olkin (KMO) value was 0.945, exceeding the threshold of 0.70. The variance explained by each factor was as follows: educational experience (11.695%), entertainment experience (23.128%), escapist experience (33.659%), aesthetic experience (43.504%), self-expansion (53.272%), place dependence (62.529%), place identity (71.060%), and eWOM intention (79.585%). These results provide initial support for the construct validity of the measurement instrument.

Confirmatory factor analysis (CFA) was subsequently conducted to evaluate the measurement model. The results suggested an acceptable model fit: *χ*^2^ = 585.858, df = 224, *p* < 0.001; *χ*^2^/df = 2.615 (below 3.0); GFI = 0.908 (> 0.90); CFI = 0.947 (> 0.90); TLI = 0.935 (> 0.90). The RMSEA was 0.063, and the RMR was 0.025, further indicating that the model corresponds well with the observed data (Table 2).

Convergent and discriminant validity were also examined. Composite reliability (CR) values for all latent constructs exceeded 0.70, and average variance extracted (AVE) values reached the recommended 0.50 level, supporting convergent validity (Fornell and Larcker, 1981). Furthermore, the square root of the AVE for each construct exceeded its correlations with other constructs, providing evidence for discriminant validity (Table 3).

**Table 3:** Results of discriminant validity (Fornell and Larcker, 1981).

Variable	CR	AVE	EDUE	ENTE	ESCE	AESE	SE	PD	PI	eWOM
EDUE	0.899	0.748	0.865							
ENTE	0.838	0.633	0.773	0.795						
ESCE	0.881	0.712	0.400	0.655	0.844					
AESE	0.851	0.655	0.458	0.612	0.766	0.810				
SE	0.834	0.627	0.652	0.783	0.685	0.627	0.792			
PD	0.831	0.621	0.688	0.704	0.481	0.459	0.680	0.788		
PI	0.911	0.774	0.553	0.737	0.708	0.674	0.736	0.782	0.880	
eWOM	0.827	0.614	0.606	0.690	0.598	0.544	0.710	0.729	0.728	0.784

CR, Composite Reliability; AVE, Average Variance Extracted. Bold values on the diagonal represent the square roots of the AVE. Discriminant validity is assessed according to the Fornell–Larcker criterion (Fornell and Larcker, 1981).

### 4.3 Structural mode

Structural equation modeling (SEM) was conducted using IBM AMOS 26 to examine the proposed research model. The structural model demonstrated an acceptable fit to the data (*χ*^2^ = 583.183, df = 224, *p* < 0.001; *χ*^2^/df = 2.603; GFI = 0.920; CFI = 0.948; IFI = 0.936; RMR = 0.029; RMSEA = 0.069), indicating that the proposed model adequately represented the observed relationships among the study variables (Table 4).

**Table 4:** Structural model results.

Hypothesis	Path	*β*	*t*-value	*p*-value	Results
H1a	EDUE → SE	0.198	2.468	*	Yes
H1b	ENTE → SE	0.396	3.747	***	Yes
H1c	ESCE → SE	0.294	3.443	***	Yes
H1d	AESE→ SE	0.069	0.921	n.s	No
H2a	EDUE → PD	0.257	3.065	**	Yes
H2b	ENTE → PD	0.308	2.621	**	Yes
H2c	ESCE → PD	−0.004	−0.041	n.s	No
H2d	AESE→ PD	−0.039	−0.501	n.s	No
H2e	EDUE → PI	−0.041	−0.561	n.s	No
H2f	ENTE → PI	0.347	3.339	***	Yes
H2g	ESCE → PI	0.175	2.178	*	Yes
H2h	AESE→ PI	0.160	3.310	*	Yes
H3a	SE → PD	0.343	3.704	***	Yes
H3b	SE → PI	0.300	3.743	***	Yes
H4a	PD → eWOM	0.459	7.094	***	Yes
H4b	PI→ eWOM	0.426	6.983	***	Yes

n.s., not supported; β, standardized regression weights; p, probability.

***p < 0.001, **p < 0.01, *p < 0.05.

The results indicated that the majority of the proposed hypotheses were supported. Specifically, educational, entertainment, and escapist experiences were positively associated with self-expansion. However, the association between aesthetic experience and self-expansion (H1d) was not statistically significant. Similarly, escapist experience was not significantly linked to place dependence (H2c), aesthetic experience showed no significant association with place dependence (H2d), and educational experience was not significantly related to place-related identity (H2e).

These non-significant results suggest that the psychological associations of tourism encounters may vary across experiential dimensions. Rather than all dimensions relating equally to self-expansion and place attachment, certain experiences appear to be more relevant for identity-related processing and emotional bonding within the ancient town context.

To further examine the proposed mediation mechanisms, a bootstrap analysis with 5,000 resamples and bias-corrected 95% confidence intervals (CIs) was conducted (Table 5). The direct associations between the four experiential dimensions and eWOM intention were not statistically significant, as all corresponding 95% CIs included zero. This indicates that tourism experiences are not directly linked to eWOM intention when accounting for the mediating roles of self-expansion and place attachment.

**Table 5:** Bootstrap estimates of direct and indirect effects.

Path	*β*	Bootstrap SE	LLCI	ULCI	Results
EDUE →eWOM (direct effect)	−0.002	0.075	−0.002	0.294	No
ENTE →eWOM (direct effect)	−0.164	0.111	−0.164	0.274	No
ESCE →eWOM (direct effect)	−0.06	0.083	−0.060	0.265	No
AESE →eWOM (direct effect)	−0.72	0.078	−0.122	0.187	No
EDUE → eWOM (total effect)	0.253	0.069	0.115	0.386	Yes
ENTE → eWOM (total effect)	0.464	0.076	0.312	0.607	Yes
ESCE → eWOM (total effect)	0.271	0.063	0.148	0.393	Yes
AESE → eWOM (total effect)	0.122	0.060	0.004	0.241	Yes
EDUE → SE → PD → eWOM (indirect effect)	0.364	0.052	0.262	0.464	Yes
EDUE → SE → PI → eWOM (indirect Effect)	0.152	0.035	0.086	0.224	Yes
ENTE → SE → PD → eWOM (indirect effect)	0.308	0.064	0.189	0.439	Yes
ENTE → SE → PI → eWOM (indirect effect)	0.280	0.050	0.185	0.381	Yes
ESCE → SE → PD → eWOM (indirect effect)	0.156	0.056	0.049	0.269	Yes
ESCE → SE → PI → eWOM (indirect effect)	0.249	0.042	0.169	0.334	Yes
AESE → SE → PD → eWOM (indirect effect)	0.037	0.056	−0.067	0.150	No
AESE → SE → PI → eWOM (indirect effect)	0.125	0.036	0.060	0.201	Yes

*β*, standardized effect; Bootstrap SE, bootstrap standard error; LLCI, lower limit of the 95% confidence interval; ULCI, upper limit of the 95% confidence interval. Yes, significant indirect/direct/total effect (95% CI excludes zero); No, non-significant effect (95% CI includes zero).

In contrast, several indirect associations were statistically significant. Specifically, serial mediation paths through self-expansion and place dependence, as well as through self-expansion and place-related identity, were significant for educational, entertainment, and escapist experiences (95% CIs excluded zero). These findings support the proposed mechanism that tourism experiences are indirectly associated with eWOM intention through their links with self-expansion, which in turn relates to place attachment and online advocacy.

Regarding aesthetic experience, the indirect association via self-expansion and place dependence was not significant. However, the path through self-expansion and place-related identity remained significant. This suggests that aesthetic encounters may be associated with eWOM intention primarily through identity-related identification with the destination rather than through functional dependence.

## 5 Discussion

This study examined an integrated model based on the Experience Economy framework and the Stimulus–Organism–Response (S–O–R) paradigm to explore how tourism experiences relate to self-expansion, place attachment, and electronic word-of-mouth (eWOM) intention. The findings provide insights into the psychological processes through which tourism encounters may become integrated into self-concepts, subsequently relating to identity-relevant responses. By examining self-expansion as a psychological mechanism, this study extends existing research beyond satisfaction-based models and contributes to a broader understanding of self-concept enrichment within experiential consumption.

First, the results indicate that educational, entertainment, and escapist experiences are positively associated with tourists’ self-expansion, while aesthetic experience did not show a significant association. These findings suggest that self-expansion may be primarily linked to experiences requiring active cognitive engagement and emotional involvement rather than passive sensory appreciation alone. In other words, perceived psychological growth appears to relate more closely to the active interpretation of experiences. This is consistent with Self-Expansion Theory, which suggests that individuals enrich their self-structures by incorporating novel perspectives and resources (Aron et al., 2013). Accordingly, tourism encounters appear to relate to self-expansion when they offer opportunities to broaden existing self-perceptions.

The non-significant association between aesthetic experience and self-expansion offers a nuanced view of the Experience Economy framework. A possible explanation lies in the increasing homogenization of ancient town tourism (Chen et al., 2023). As environments become standardized, aesthetically pleasing settings may no longer provide the novelty necessary to stimulate identity-related processing. Consequently, while aesthetic encounters may relate to temporary enjoyment, they may be insufficient to facilitate the deeper self-concept enrichment associated with self-expansion. This suggests that psychological processes in tourism may depend on the perceived uniqueness and personal significance of the encounter.

Second, educational and entertainment experiences were associated with both place dependence and place-related identity, whereas escapist and aesthetic experiences showed limited associations with place attachment. These results suggest that attachment may develop through experiences that facilitate meaning-making and emotional engagement. Educational encounters may relate to functional attachment by enhancing the perceived value of the destination, while entertainment relates to positive affect that reinforces emotional bonds.

In contrast, escapist experiences may relate only to temporary psychological detachment without creating enduring destination-specific meanings. Similarly, the role of aesthetic experiences may be diminished when visually similar environments reduce the perceived distinctiveness of a destination. Notably, the lack of a significant link between educational experience and place-related identity suggests that acquiring knowledge alone may not suffice for a destination to become integrated into the self-concept. Instead, identity-related attachment may require affective engagement that individuals internalize as part of their evolving identities.

Third, consistent with the proposed framework, self-expansion showed a positive association with both place dependence and place-related identity. This supports the perspective that tourists may develop attachment not only because a destination satisfies functional needs, but because the experiences become incorporated into their self-concepts. From a personality and social psychology lens, these findings suggest that self-expansion may function as a mechanism through which meaningful experiences relate to the development of enduring psychological bonds with specific environments. Rather than merely being an outcome, self-expansion explains why certain encounters acquire symbolic significance and subsequently relate to emotional and behavioral responses.

Finally, the results indicate that both place dependence and place-related identity are associated with tourists’ eWOM intention. This suggests that tourists are more likely to engage in online advocacy when they perceive a destination as personally significant. Rather than a mere post-consumption evaluation, eWOM may represent a form of identity expression through which individuals communicate experiences integrated into their self-structures. By sharing meaningful encounters via digital platforms, tourists may reinforce their own identities while simultaneously advocating for the destination. This interpretation extends previous eWOM research by suggesting that online advocacy may be associated with identity-related psychological processes emerging from meaningful tourism encounters.

### 5.1 Theoretical implications

The present study offers several theoretical insights by examining the associations between tourism experiences, self-concept enrichment, and identity-related responses. Notably, it contributes to personality and social psychology by positioning tourism as a context for self-construction and identity performance.

First, this study extends the application of Self-Expansion Theory within heritage tourism. While prior research has viewed destinations as symbolic brands, this study suggests that tourism encounters—specifically those requiring active cognitive and emotional engagement—are associated with the integration of novel resources into the self-structure. From a PSP perspective, the findings indicate that self-expansion is not merely a post-consumption outcome but a dynamic psychological process through which individuals enrich their self-concept breadth. This highlights that tourism experiences relate to personal growth when they provide opportunities for individuals to incorporate new perspectives and identities into their evolving self-structures (Aron et al., 2013).

Second, this study enriches the literature on place attachment by exploring the link between self-concept inclusion and place-related identity. By integrating the S–O–R framework with Self-Expansion Theory, the findings provide a psychological explanation for how a destination moves from being a mere physical setting to becoming a part of the extended self. This suggests that place-related identity is associated with the extent to which the destination’s symbolic meanings are internalized during the self-expansion process. In doing so, the study extends existing knowledge by demonstrating that attachment to culturally significant environments may reflect a psychological state where the destination is integrated into one’s identity-related memories and self-perceptions.

Third, the proposed framework contributes to the understanding of electronic word-of-mouth (eWOM) as a form of identity expression and self-presentation. Moving beyond satisfaction-based models, this study suggests that the intention to engage in online advocacy is associated with the psychological significance of the experience to the individual’s self-concept. From a social psychological lens, sharing travel encounters on digital platforms may represent a performance of the “expanded self,” where tourists signal their enriched identities and social resources to an audience. By positioning eWOM as a medium for identity-relevant communication, this study offers a more nuanced perspective on why certain tourism experiences relate to voluntary online advocacy in contemporary digital environments.

### 5.2 Practical implications

The findings of this study offer several practical implications for the sustainable management of ancient town tourism destinations.

First, the results suggest that educational, entertainment, and escapist experiences are more closely associated with tourists’ self-expansion than aesthetic encounters. This indicates that destination managers should move beyond merely enhancing visual attractiveness and instead design tourism encounters that facilitate active cognitive and emotional engagement. As many ancient towns face increasing homogenization, management should prioritize creating distinctive, participatory cultural experiences that encourage learning and personal reflection. For instance, immersive storytelling, folk craft workshops, and hands-on heritage activities provide opportunities for tourists to actively engage with local culture, which may relate to self-concept enrichment. Such psychologically significant experiences are likely to differentiate destinations more effectively than aesthetic improvements alone.

Second, because educational and entertainment encounters are associated with place attachment, destination managers should emphasize interactive cultural interpretation over passive information delivery. Heritage programs should encourage active participation rather than mere observation. Interactive museums, intangible cultural heritage workshops, and immersive performances may relate to stronger emotional bonds by facilitating cognitive understanding and emotional engagement. Furthermore, digital technologies such as augmented reality (AR) and virtual reality (VR) can be integrated into heritage interpretation to enrich visitor experiences. Rather than replacing authentic cultural encounters, these technologies should be utilized to deepen visitors’ understanding of local history and culture, thereby increasing opportunities for meaningful psychological participation.

Third, the findings suggest that place dependence and place-related identity are positively associated with tourists’ eWOM intention. Destination managers should, therefore, view eWOM not merely as a marketing outcome but as a reflection of psychologically significant tourism experiences. Instead of encouraging visitors to share superficial photographs, managers should facilitate experiences that tourists voluntarily share as a form of identity expression. Emotional storytelling and personalized cultural encounters may relate to tourists’ desire to communicate their experiences through social media. Such user-generated content, perceived as highly credible, can contribute to the long-term sustainability and competitiveness of ancient town tourism by signaling identity-related satisfaction to potential visitors.

Overall, the present findings suggest that the management of ancient towns should focus not only on preserving physical landscapes but also on designing experiences that relate to self-expansion and emotional attachment. By creating culturally authentic and psychologically meaningful encounters, destination managers can enhance visitor well-being, strengthen emotional bonds with the destination, and encourage online advocacy.

### 5.3 Limitations and future research directions

Despite its theoretical and practical contributions, this study has several limitations that suggest directions for future research.

First, the use of a cross-sectional survey design limits the ability to draw causal inferences regarding the associations between tourism experiences, self-expansion, place attachment, and eWOM intention. Although the model is theoretically grounded in the S–O–R framework, the results should be interpreted as associative rather than causal. Future studies are encouraged to adopt longitudinal or experimental designs to examine how identity-related psychological responses evolve over time and to provide stronger evidence for the directionality of these links.

Second, the data were collected using single-source, self-reported questionnaires, which may be subject to common method bias (CMB) and social desirability bias. While procedural remedies and Harman’s single-factor test suggested that CMB was not a major concern, it cannot be entirely ruled out in a cross-sectional self-report study. Future research should consider more rigorous statistical approaches, such as marker-variable techniques or full collinearity VIF assessments. Additionally, combining questionnaire data with digital trace data (e.g., social media analytics or GPS tracking) could further enhance the robustness of the findings.

Third, the study focused exclusively on five ancient water towns in the Jiangnan region of China. While this context is appropriate for examining heritage tourism, the external validity of the findings across other cultural settings remains limited. Future research should examine the proposed framework in diverse environments—such as rural tourism, national parks, or international destinations—to evaluate its cross-cultural applicability and generalizability.

Finally, this study examined direct and mediating associations without considering potential boundary conditions. From a personality and social psychology perspective, future research could investigate the moderating roles of individual psychological characteristics, including personality traits (e.g., openness to experience), travel motivations, and cultural identity. Exploring these moderators would provide a more nuanced understanding of the psychological processes through which tourism encounters relate to self-concept enrichment and subsequent behavioral responses across different individual profiles.

## Data Availability

The original contributions presented in the study are included in the article/Supplementary material, further inquiries can be directed to the corresponding author.
